# The Social Lives of Infectious Diseases: Why Culture Matters to COVID-19

**DOI:** 10.3389/fpsyg.2021.648086

**Published:** 2021-09-23

**Authors:** Rebeca Bayeh, Maya A. Yampolsky, Andrew G. Ryder

**Affiliations:** ^1^Culture, Health, and Personality Lab and Centre for Clinical Research in Health, Department of Psychology, Concordia University, Montreal, QC, Canada; ^2^School of Psychology, Université Laval, Montreal, QC, Canada; ^3^Culture and Mental Health Research Unit and Lady Davis Institute for Medical Research, Jewish General Hospital, Montreal, QC, Canada

**Keywords:** COVID-19, pandemic, cultural psychology, cultural-clinical psychology, infectious disease, racism, discrimination

## Abstract

Over the course of the year 2020, the global scientific community dedicated considerable effort to understanding COVID-19. In this review, we discuss some of the findings accumulated between the onset of the pandemic and the end of 2020, and argue that although COVID-19 is clearly a biological disease tied to a specific virus, the culture–mind relation at the heart of cultural psychology is nonetheless essential to understanding the pandemic. Striking differences have been observed in terms of relative mortality, transmission rates, behavioral responses, official policies, compliance with authorities, and even the extent to which beliefs about COVID-19 have been politicized across different societies and groups. Moreover, many minority groups have very different experiences of the pandemic relative to dominant groups, notably through existing health inequities as well as discrimination and marginalization, which we believe calls for a better integration of political and socioeconomic factors into cultural psychology and into the narrative of health and illness in psychological science more broadly. Finally, individual differences in, for example, intolerance of uncertainty, optimism, conspiratorial thinking, or collectivist orientation are influenced by cultural context, with implications for behaviors that are relevant to the spread and impact of COVID-19, such as mask-wearing and social distancing. The interplay between cultural context and the experience and expression of mental disorders continues to be documented by cultural-clinical psychology; the current work extends this thinking to infectious disease, with special attention to diseases spread by social contact and fought at least in part through social interventions. We will discuss cultural influences on the transmission, course, and outcome of COVID-19 at three levels: (1) cross-society differences; (2) within-society communities and intergroup relations; and (3) individual differences shaped by cultural context. We conclude by considering potential theoretical implications of this perspective on infectious disease for cultural psychology and related disciplines, as well as practical implications of this perspective on science communication and public health interventions.

## Introduction

In 2020, the global scientific community published more than 70,000 articles on COVID-19 (Pujol, 2020). Most of this work emphasized the biology of the virus and mechanisms underlying its transmission, as well as characteristics of the resulting disease, treatment alternatives, and vaccine development. Some attention has also been paid to behavioral reactions to the pandemic, as well as to several social, political, and psychological influences on its development in different societies. The overarching aim of this review is to argue that this emerging body of research is not only relevant to understanding how COVID-19 is shaped by the mutual constitution of culture and mind, but also points toward a framework upon which a cultural perspective on infectious diseases can be built. In short, we believe that cultural psychology has much to offer to the study of infectious disease.

Our choice to cover the first year of the pandemic highlights the early contributions of cultural psychological research—and places some practical, albeit artificial, limits on this rapidly proliferating literature. Of course, the pandemic situation continues to evolve. As new variants emerge and as vaccines are deployed across the globe, our understanding of the interplay between culture and COVID-19 will continue to grow. Future findings might bolster, contradict, or complicate our conclusions and proposed framework. Moreover, not only might our conclusions change as we collect more and better data, the pandemic marks a period of rapid cultural–historical change. As such, observed relations between variables may well shift over time, not least as policy makers, journalists, scientists, and the general public respond to the ever-growing database about COVID-19. We believe that cultural psychology has established the empirical tools to conduct research that is both rigorous and time-sensitive, while also offering the conceptual tools to help us better understand how and why the picture may shift over time (see Gergen, 1973).

While most published research on behavioral responses to COVID-19 addresses local contingencies, particularly in the US, we will expand some of the recently proposed pandemic responses into a broader, global perspective. Our primary aim is to analyze the pandemic not only in terms of previously established and widespread paradigms in the fields of cultural and cultural-clinical psychology, but also to explore potential contributions that these fields have to offer to our understanding of infectious disease, by enabling a deeper analysis of how the mutual constitution of culture and mind are influenced by larger socioeconomic dynamics. We also hope to promote dialogue with researchers from other fields (e.g., health psychology, medical anthropology) that could benefit from these perspectives. Traditional health sciences have often assessed local expressions of health and illness from an analytical, Western perspective. The meta-theoretical perspective of cultural psychology expands our ability to interpret such expressions by encouraging reflection on how science-making might, in itself, constitute a manifestation of a culturally-biased way of understanding human behavior (see also Hoshmand, 1996; Adams and Salter, 2007).

The research question guiding this paper is: *how can cultural psychology and cultural-clinical psychology help us understand the global development of the pandemic, given what we learned in 2020?* We will cover literature from around the world, including regions of the globe not represented as frequently as the Global North including, where feasible, literature produced in countries that are not traditionally science-exporting (see Adams and Salter, 2007). We will first introduce cultural and cultural-clinical psychology, and how they might be brought to bear on infectious disease. Then, we will consider three levels at which culture and mind interrelate in the context of the pandemic: (1) across societies, where “country” or “region” is the unit of analysis and used as a proxy for “culture;” (2) within a given country or region, where cross-cultural variation is observed across ethnocultural communities and in the relations between them; and (3) in individual people, with variation across person-level characteristics that are shaped by culture. We will then examine the theoretical and practical implications of a cultural psychological perspective on infectious disease.

## Cultural and Cultural-Clinical Psychology Perspectives

Several subdisciplines of psychology concern themselves with the relation of the human mind and behavior with its sociocultural context. Here, we take a “big tent” and pluralistic approach to cultural psychology and include not only the various traditions under that name, but also cross-cultural psychology, ethnic minority psychology, and other subdisciplines within the social sciences with similar concerns (e.g., psychological anthropology, cognitive sociology). This is aligned with the interdisciplinary spirit of cultural psychology, especially vital links with anthropology (Shweder, 1991; Chirkov, 2016).

Nonetheless, we do place certain core ideas from cultural psychology at the center of our understanding. First, we adopt a working definition of culture as a set of meanings (i.e., values, beliefs, knowledge, norms) that are required to function in a particular community (Goodenough, 1994). Second, we hold that cultural meanings are observable in the world as consensually-understood practices and products that emerge from and support these meanings (Ryder et al., 2011; Morling, 2016). Third, we follow Shweder (1991) in understanding culture and mind as existing in a relation of mutual constitution; we cannot fully understand one without referring to the other. The emergence of cultural neuroscience and neuroanthropology expanded this understanding to the human brain: culture, mind, and the brain “make each other up” (Kitayama and Uskul, 2011; Ryder et al., 2011). Fourth, we center ourselves in the conviction that people *should be understood* in context but *should not be reduced* to their contexts, and especially not to generalized stereotypes of their cultural groups. Thus, individual differences in cultural psychology represent much more than mere “dispositional tendencies;” rather, they are a manifestation of each person's history of engagement with their available cultural affordances (Adams, 2012) and, as we argue here, the sociopolitical dynamics of power that permeate cultural ecologies.

Although group differences can highlight ways in which very different cultural contexts shape experience and behavior, we must keep in mind that every aggregate score conceals numerous individual people who might conform to, resist, amplify, deliberately rebel against, or simply vary from the local consensus. Related to this perspective is cultural psychology's emphasis on “unpacking culture”: starting by cataloging observations to be sure but moving toward explanations of why cultural group differences are observed (Heine and Norenzayan, 2006). Note that these explanations also leave room for individual variability. For example, if a group difference in health outcomes is due to wealth disparity, we can better understand how unusually wealthy members of the disadvantaged group might have better outcomes, rather than simply considering them as outliers.

### Theoretical Insights From Cultural-Clinical Psychology

Cultural-clinical psychology has emerged over the past decade at the intersection of cultural psychology and clinical psychology, applying a cultural lens to psychopathology and its treatment (Ryder et al., 2011; Chentsova-Dutton and Ryder, 2019). In keeping with the interdisciplinary spirit of cultural psychology, cultural-clinical psychology interfaces with a number of adjacent disciplines—including several health-related disciplines that are less well-known to cultural psychologists, such as cultural psychiatry or the social sciences of medicine. Although COVID-19 is a viral infection, there are certain parallels that warrant a closer look at this perspective. In particular, the social transmission of infectious diseases means that their spread, and hence their impact on a population, is driven in part by social behaviors which, in turn, are shaped by patterns of culturally shared beliefs in that population. This perspective has parallels in social psychological approaches that have proven useful in public health, such as the *theory of planned behavior*, in which personal attitudes, perceived social norms, and sense of control combine to predict health-relevant behavioral intentions (e.g., Godin and Kok, 1996; Montaño and Kasprzyk, 2015).

Cultural-clinical psychologists have adapted a tripartite understanding of *disorder* from the social sciences of medicine, one that maps closely onto the distinction between culture, mind, and brain. In this view, there is an important distinction between disease and illness, where the disease is the biological dysfunction and the illness is the sufferer's subjective experience of that disorder (Boorse, 1975; Eisenberg, 1977). A third term, *sickness*, is then used to talk about the social context in which this experience takes place (Twaddle, 1973). Even when the researcher has chosen to narrow in on a particular aspect of a disorder—for example, how the novel coronavirus interacts with lung tissue (disease), or the subjective experience of determining whether one's breathing problems are sufficient to warrant a hospital visit (illness), or local beliefs that stigmatize the recovering patient as having been careless or unclean (sickness)—the larger goal ought to be incorporation of these findings into an integrated scientific narrative.

### Looping Effects

Attending to psychological and sociocultural aspects in ways that emphasize how they interrelate with each other and with biology opens possibilities for thinking about the cultural psychology of viral diseases. In the study of culture and mental health, the idea of *looping effects* has helped to clarify how beliefs can interact with physiological processes to yield strikingly different symptom presentations in different cultural contexts (Kirmayer and Sartorius, 2007; Ryder and Chentsova-Dutton, 2015). Hacking (1995) originally proposed this idea to help explain how the definitions of human-made categories inevitably shift over time as a result of the feedback loops that emerge when people learn about, discuss, debate, contest, and otherwise respond to the categories themselves. Cultural psychiatrists and cultural-clinical psychologists have since pushed this concept to show how similar looping patterns can yield different categories of disorder in different cultural contexts.

Consider Clark's (1986) model of a panic attack. A person detects a sensation such as chest pain or accelerated heart rate. As they hold a belief that such experiences might signal heart trouble, they grow concerned and focus more on these sensations. The mounting anxiety is accompanied by arousal of the autonomic nervous system, which generates a number of additional sensations—including chest pain and accelerated heart rate. However, people can hold very different beliefs about what different sensations might signal. In Cambodia, for example, local beliefs about the body lead some people to become alarmed by neck stiffness or flushing, sensations that can also be exacerbated by autonomic arousal (Hinton et al., 2001). The shared biology of the autonomic nervous system combines with culturally-shaped beliefs to produce very different symptom presentations, albeit presentations with a common core (Ryder and Chentsova-Dutton, 2015). In other words, different symptomatologies and diagnostic categories emerge in different cultural contexts *because of* universal looping processes.

Looping effects have not yet been extensively explored for infectious diseases from a psychological perspective, although similar patterns have been observed by medical anthropologists (e.g., Alenichev, 2021). People's beliefs do not act directly on the virus itself and are not so clearly implicated in the core symptoms of the infection. Yet individually- and especially collectively-held beliefs can change important features of a socially transmitted disease, such as infection rate. Indeed, the virus itself can be changed by these processes insofar as certain practices increase spread, providing more opportunities for random genetic mutations. More predictable are the effects of particular beliefs about risk, spread, impact, and protective measures, set against the backdrop of more general beliefs about how to balance health and the economy, whether or not to trust political or medical authorities, ingrained customs such as mask-wearing when feeling ill or greeting people with a handshake, and so on.

Here, looping effects can be examined at the level of individual people: for example, if individualistic values in a given cultural context are associated with higher perceived danger and lower trust in authority, that could increase fear—and if increased fear leads to more conspiratorial thinking and reduced willingness to take protective measures, infection rates could worsen, leading to more fear, perhaps even less trust of the authorities reporting those rates, and so on. These looping effects can also be examined within social networks, and that might be the most important level for a socially-transmitted disease. A single person with a given belief will not make much difference to the spread of infectious disease, but widespread consensus around certain beliefs could do so. Often, in such cases, culturally-shaped values and beliefs are fundamentally rooted in political and socioeconomic conditions. For example, discrimination against devalued groups combined with stigma that includes beliefs about the disease-proneness of those groups could lead to worse health services in certain neighborhoods and less trust in health authorities. Those effects could lead to higher rates and greater symptom severity in devalued groups, which in turn would reinforce the “disease-prone” stereotypes. This latter example fits with work in population and public health on *syndemics*, in which the impact of a disease intersects with the impact of social disadvantage to create very different illness experiences (Singer et al., 2017).

### Cultural Scripts

So far, we have discussed “beliefs” in general terms. In cultural-clinical psychology, however, beliefs can be understood in a systematic way, as aspects of *cultural models*, clusters of consensually-understood assumptions with associated behaviors in the form of specific *cultural scripts*. These ideas are derived from work in cognitive science: consider the classic example of the restaurant script, in which communities hold shared assumptions about the sequence one generally follows when booking a table, checking one's coat, being seated, reading the menu, and so on (Schank and Abelson, 1977). Cultural scripts change over time and mutually constitute individuals' relations with their groups, their environment, and their bodies. In the health domain, general *cultural models for normalcy and deviancy* can be invoked to organize the beliefs one has about optimal, normal, suboptimal, and pathological functioning (Chentsova-Dutton and Ryder, 2020). Although this approach has to date been applied mainly to mental disorders, it can be extended to physical health and illness. Cultural models for health and illness, along with scripts for specific disorders (such as COVID-19), establish “thresholds of concern” that help people make decisions. For example: what sensations should I monitor? What signals that I might be ill? What would the recovery process look like? In the case of an emerging condition such as COVID-19, these scripts build on previous understandings and experiences of infectious diseases, but can also shift rapidly as more is learned.

## Cross-Societal Differences: Cultural Values and Relational Mobility

One way of understanding the interplay between culture and mind in the context of infectious diseases is to analyze outcomes across different countries or regions. Cross-cultural psychologists have developed methods that allow comparisons of large numbers of societies. In such studies, each society—generally a country or an autonomous territory—is treated as a separate observation. Large-scale studies of personality traits or values, for example, yield society-level averages for traits such as extraversion or values such as individualism-collectivism. These sets of averages can then serve as indices for future studies conducted at the cross-societal level.

Murray and Schaller (2010) published an index of historical pathogen prevalence to help researchers evaluate the extent to which geographical variation in infectious disease risk may have helped to shape cultural variations. Following the *parasite-stress model of human sociality*, researchers have predicted that cultural contexts in regions with a history of higher risk for infectious diseases would develop social practices that limit encounters with strangers. Several studies have shown that higher levels of historical pathogen prevalence are associated with lower levels of extraversion and openness-to-experience (Schaller and Murray, 2008) as well as lower levels of individualistic (vs. collectivistic) values (Fincher et al., 2008) and conformity (Murray et al., 2011), all of which serve to limit extensive social contacts with outgroup members.

Cross-cultural studies are not without limitations. While some countries are developing their own technologies to monitor and analyze data on COVID-19, others still rely on external help with tests, studies and medical equipment (Renzaho, 2020). Furthermore, the reliability of data is lower in poorer countries (Muurlink and Taylor-Robinson, 2020) and in countries where COVID-19 infections were seriously under-reported, such as Brazil (Freire, 2020; Silva and Figueiredo Filho, 2020), China (Colson, 2020), the US (van Beusekom, 2020) and Russia (Kofanov et al., 2020; Nechepurenko, 2020). As these limitations pose challenges to the development of more comprehensive cross-societal research, we believe these data collection disparities should continue to be substantially integrated into cross-cultural studies on infectious diseases.

### Cultural Values: Individualism-Collectivism and Tightness-Looseness

Cultural values reflect widely shared priorities that can influence, for instance, specific beliefs about illness, conformity, and how to balance health and economic concerns. There is a considerable amount of research on this topic, focusing especially on individualism-collectivism. Recent years have also seen growing interest in tightness-looseness. Both dichotomies have been studied in relation to COVID-19.

Individualistic cultural contexts, as established in the cultural psychology literature, tend to prioritize an independent construal of self, as well as freedom and fulfillment of personal goals; conversely, in collectivistic societies, group ties and responsibilities are perceived more important (Triandis et al., 1986; Markus and Kitayama, 1991). In the context of the pandemic, in individualistic societies, governments might be more hesitant to take compulsory measures in response to COVID-19, such as lockdowns and mandatory mask-wearing, resulting in delayed responses to public health emergencies. Moreover, people in those societies might be less accustomed to following public health recommendations for common infectious diseases (e.g., wearing a mask whenever one has cold or flu symptoms).

Indeed, between March and May of 2020, the relative number of COVID-19 cases and deaths in more individualistic countries were considerably higher (Jiang et al., 2020). In a more recent study comparing data from 98 countries up to July of 2020, collectivism was associated with fewer cases and fewer deaths per million people cross-culturally, although GDP per capita played a stronger role in predicting those variables (Webster et al., 2021), highlighting the importance of the interplay between cultural and socioeconomic considerations in research and public policies. More recently collected data demonstrated that, by August of 2020, the fatality rate was still higher in more individualistic countries (Melton, 2020, preprint). Curiously, in a cross-state analysis in the US (a country with a highly individualistic orientation that was seriously affected by COVID-19), Webster et al. (2021) found that collectivistic states had higher case and death rates, but the racial-ethnic composition of the states were stronger predictors of both. Thus, systemic racial health disparities might play a stronger role in predicting the impact of COVID-19 than collectivism.

Individualism and collectivism have been linked with how people conceptualize the ontological reality of health and illness. In Western-educated and individualist populations, medical models are based on *analytic thinking* (Choi et al., 2007), which is characterized by examining each component of a system as discrete and independent from the whole, as well as zero-sum reasoning (e.g., “if X exists, then Y cannot”) (Nisbett et al., 2001). Medical approaches based on this model tend to frame illness as an intrusion within an inherently independent body, and home in on a specific set of symptoms or a specific disorder (Good, 1993; Jayasundar, 2010). Conversely, *holistic thinking*, which is found in populations with greater collectivism and without Western-based education, is characterized by examining the relationship between each element in a system to form the whole, where multiple pieces can simultaneously exist while moving in different directions or serving different functions (Nisbett et al., 2001). Holistic medical systems, such as Ayurveda (Jayasundar, 2010), Traditional Chinese Medicine (Koo and Choi, 2016), and multiple Indigenous medical systems (Dahlberg and Trygger, 2009; Auger et al., 2016) account for illness as an imbalance in functioning within the individual's whole physical and psychological system, and within the community and broader environmental context (Dahlberg and Trygger, 2009; Jayasundar, 2010; Koo and Choi, 2016). As a result, the frame that individuals use to understand and address COVID-19 shifts according to the cultural reasoning style and associated medical model. To illustrate, while also supporting vaccinations, Ayurvedic doctors and researchers have examined COVID-19 in terms of how the disease functions within the larger weakened system, proposing that treatment should focus on ensuring that global bodily functions are supported while strengthening immune and respiratory systems to prevent infection, and to ease the course of the disease should one contract the virus (Niraj and Varsha, 2020; Rajkumar, 2020; Rastogi et al., 2020).

Finally, tightness-looseness, a country-level measure of the strictness of societal rules, may be related to the formal and informal enforcement of, and consequent adherence to, restrictive measures such as social distancing and stay-at-home orders. Tighter societies might, for instance, enforce lockdown measures with less tolerance of non-adherence, while looser societies might adopt more lax regulations, contributing to viral spread. Cao et al. (2020) examined correlations between the impact of the pandemic in 54 different countries and the interplay between individualism-collectivism and three different indexes of tightness-looseness. The increase of reported cases and deaths per million inhabitants, as well as the case fatality rate, were all positively associated with greater individualism and looseness. Moreover, countries that experienced the most severe increases of deaths per million in the population between the 16th and the 45th days of the implementation of lockdowns had a combination of higher individualism and higher looseness (Cao et al., 2020).

### Relational Mobility

The degree to which interpersonal relationships are fixed or voluntary in a given group or society has been defined as *relational mobility* (Thomson et al., 2018). Higher relational mobility entails greater choice among numerous new connections in frequently-changing social circles, and is usually associated with geographical mobility, whereas lower relational mobility involves fewer options for new connections with others, and instead revolves around maintaining existing connections in smaller, more stable social circles. On a historical scale, cultural variation in relational mobility is rooted in histories of subsistence farming structures, with higher relational mobility associated with more independent and mobile subsistence styles (Thomson et al., 2018).

In the context of infectious diseases, relational mobility might be particularly relevant to the likelihood of potential carriers of the novel coronavirus traveling across different geographical areas, increasing the probability of new cases emerging in different regions. Indeed, by using country-level scores for relational mobility (Thomson et al., 2018) and publicly available pandemic data from Johns Hopkins University (Center for Systems Science Engineering (CSSE) at Johns Hopkins University, 2020), Salvador et al. (2020) analyzed the correlations between relational mobility and the number of COVID-19 cases and deaths in the first 30 days of the outbreak across 39 different countries. The growth of both the number of cases and the number of deaths was significantly faster in countries with higher relational mobility. This difference suggests that sociocultural ecologies in which individuals are less reserved and more likely to make acquaintances outside of their primary social groups are more vulnerable to the spread of COVID-19.

## Within-Society Intergroup Relations

By exploring how different groups within the same society experienced, reacted to, and were impacted by the pandemic, we can endeavor to integrate a cultural psychology perspective with a broad set of political and socioeconomic considerations. We will briefly review how different age groups and genders were affected in different countries. Then, we will analyze how dynamics of polarization, power, oppression, and privilege, which are profoundly interconnected to cultural scripts, influence the outcomes of COVID-19 in different groups.

The impact experienced by different communities affects the COVID-related risk perception within those groups through social amplification. Those who had direct experience with the virus or heard about the virus from friends and family tend to perceive more risks associated with COVID-19 (Dryhurst et al., 2020). People trying to interpret a new and ambiguous situation tend to look for information about what to believe among their close contacts (Biron et al., 2020), which makes the drastic changes imposed by the pandemic a favorable scenario for social contagion of behaviors and beliefs. Normative behavioral patterns within particular social networks affect the transmission of infectious diseases, as discussed, and so do ideas about the disease's causes, consequences, protections, and treatments, especially in cases where the specific cultural scripts are still taking shape—and particularly in societies where such beliefs have been extensively politicized, which we will discuss below. Transmission of beliefs and behaviors can take place through conversation or observational learning (Debiec and Olsson, 2017) but also through traditional news sources or social media (Collinson et al., 2015; Kilgo et al., 2018; Taylor, 2019). Moreover, the centrality of social connectedness is lived out through participation in communal events, such as festivals, weddings, and funerals. Furthermore, public health authorities might be required to impose measures that directly contradict these local cultural imperatives. Impeding worship services and especially the burial of the dead can be emotionally charged for many cultural groups and communities (Schoch-Spana, 2004; Baum et al., 2009). Travel restrictions can also be burdensome on immigrant communities that are prevented from unifying with their families or attending funerals of friends and family members in their countries of origin.

Overall, members of cultural minority groups may experience numerous stressors that are not encountered by dominant groups (Taylor, 2019). These stressors are greatly exacerbated by prejudice and discrimination, which we will discuss in detail below. Even in the absence of discrimination, some minority group members such as recent migrants may experience additional obstacles (Kirmayer et al., 2011). For example, there may be linguistic barriers to effective health communication by public health officials or medical professionals (Brisset et al., 2014; Doucerain et al., 2015; Zhao et al., 2019). Unfamiliarity with how to access community resources or ongoing visa status concerns can add to the stress burden. Under pandemic conditions, there may be additional problems in accessing medical services, information about constantly-changing local regulations, or government programs for financial relief, resulting in greater psychosocial consequences (Taylor, 2019).

### Cultural and Demographic Intersections

Intersectionalities between cultural and other socio-demographic categories, such as gender, age, and social class, also affect contagion rates of infectious diseases, as well as the number of reported cases across different countries. In the case of dengue in Southeast Asia, for instance, Anker and Arima (2011) observed more reported cases in males older than 15 years of age compared to other gender-by-age groups. This difference might be linked to local cultural scripts that favor the exposure of men to mosquitoes during daytime hours. Stratifying data across different ages and genders provides us with insights on how cultural characteristics contribute to how likely women, men and families are to be exposed to the infectious diseases in different societies, as well as how likely affected individuals are to look for medical care when experiencing symptoms.

Intersections of gender with different cultural practices also produced different outcomes not only for COVID-19 risks but also to how sex and gender have shaped distress during the pandemic. Across the globe, more men than women have been dying of the disease; whereas some researchers argue this finding is more related to behavioral factors than to biological differences (Galasso et al., 2020; Pujol, 2020), others argue that women have a better immune response to the virus (Takahashi et al., 2020; Zeng et al., 2020). In the behavioral sphere, gender differences might be related to local cultural and religious norms. Muurlink and Taylor-Robinson (2020) argue that the adoption of niqabs or burkas by women in more conservative Muslim cultures might work as a protective factor against contamination through face-touching. Conversely, cultural preferences for facial hair in men might increase the risk of exposure to the virus, by compromising the seal of face coverings (Muurlink and Taylor-Robinson, 2020). Furthermore, gender segregation in some communities (e.g., Amish, Orthodox Jews) along with differing levels of involvement in different spheres of society (e.g., representation gaps in specific occupations) might also interfere with the likelihood of exposure and contamination of COVID-19 (Muurlink and Taylor-Robinson, 2020). In Panama, Peru and Colombia, policies have attempted to promote social distancing by restricting the access to services upon a gender-based rotation. This has disproportionally impacted transgender populations (Perez-Brumer and Silva-Santisteban, 2020). Overall, sexual and gender minorities have experienced more coronavirus-related physical symptoms and more depression and anxiety symptoms since the emergence of the pandemic (Moore et al., 2021).

In the mental health domain, age has yielded unexpected research findings. Older adults are known to be physically more vulnerable to COVID-19 (Crimmins, 2020; Dowd et al., 2020), which led to more social isolation in order to protect this population from infection, and sparked ageism and segregation of older adults (Lichtenstein, 2021). While the isolation experience of elderly populations was expected to produce worse mental health outcomes (Vahia et al., 2020), in several countries, the levels of stress reported by younger people were consistently higher (Kowal et al., 2020). Nonetheless, differences in mental health outcomes among seniors have been observed cross-culturally. Across the 62 nations studied by Kim and Jung (2020), older adults were more distressed in countries whose state capacity was more fragile, as measured by the Fragile State Index compiled by The Fund for Peace (2020). This index is based on the assessment of elements such as extensive corruption, involuntary dislocation of the population, economic decline, institutionalized discrimination, and group-based inequality.

Finally, different social classes, historically linked to racial and ethnocultural power dynamics as discussed in upcoming sections, are related to the likelihood of death by COVID-19 due to comorbidities (e.g., heart and liver diseases) (Marmot and Allen, 2020), and presented different chronological patterns of transmission of the virus. In countries like Germany, England, the US (Berkessel et al., 2021, preprint) and Brazil (Magenta, 2020), although richer regions and social classes were affected primarily at the beginning of the pandemic, the virus spread more quickly among poorer populations in later phases and presented higher fatality rates. These findings might be connected to access to healthcare and viability of social distancing, topics to which we will return.

### Political Polarization and the Pandemic

In some countries, the pandemic has been heavily politicized. Identification with political parties or ideologies was, at least to some extent, artificially associated with specific attitudes toward the pandemic, in turn influencing cultural scripts bound to political orientation and compliance with public health recommendations.

In Brazil, official government propaganda encouraged Brazilians to continue their routines normally. President Bolsonaro openly promoted conspiracy theories, providing misleading information about symptoms, treatments, and the severity of the pandemic in the country. These measures motivated the sector of society politically aligned with Bolsonaro, including physicians, to ignore global public health recommendations, and to acquire and utilize hydroxychloroquine, a substance used to treat malaria and other conditions with no proven efficacy against COVID-19. This resulted in several deaths and the shortage of hydroxychloroquine, depriving patients with other diseases of access to needed medication (Biller et al., 2020; Ponce, 2020; Ricard and Medeiros, 2020).

In the US, where the political system has been historically structured on a two-party basis, responses to COVID-19 have been extensively associated with partisanship. Studies using geolocation technology and debit card transaction data demonstrated that residents of Democratic counties (as determined by the 2016 presidential election) were more likely to adhere to stay-at-home orders and switch to online shopping (Painter and Qiu, 2020). Republican counties exhibited comparatively less mask use (Milosh et al., 2020), less physical distancing, and higher fatality rates (Gollwitzer et al., 2020). Behaviors and beliefs about COVID-19 are also demonstrably influenced by media consumption. Democrats frequently watch channels such as MSNBC and CNN, which recommended precautionary measures against the transmission of the disease during 2020; as a result, viewers were more likely to perceive risks associated with COVID-19, to adopt preventive behaviors and to be concerned about early lifting of government restrictions. Conversely, Republicans more frequently watch channels like Fox News, which openly and repeatedly downplayed the severity of the pandemic during 2020; viewers therefore perceived less risk associated with the pandemic and adopt fewer precautionary measures (Bruine de Bruin et al., 2020). Greater consumption of Fox News was also linked to higher reported infection and fatality growth rates (Gollwitzer et al., 2020). The politicization of the beliefs about the pandemic has also been observed in less polarized countries, such as Canada, where “anti-lockdown” parties were organized with allegedly political motivations (Keyes and Caruso-Moro, 2020).

The interaction between political polarization and attitudes toward COVID-19 can also be understood in light of Hacking's looping effects, if we consider political groups as artificially-created categories of identities that enable intentional ways of acting (see also Vesterinen, 2020). When local political categories are tied to specific beliefs and attitudes toward COVID-19, they induce self-identified members of such categories to behave in accordance with their groups.

### Discrimination, Social Inequality, Racism and Marginalization

Disease-related fears and the stigmatization of ethnocultural minorities have a long relationship. In the US, discrimination against minorities and marginalized groups has been observed across different historical epidemics: in 1892, the outbreaks of typhus fever and cholera in New York were attributed to Russian Jewish immigrants; in 1900, the bubonic plague in San Francisco was associated with Chinatown; in 1993, the hantavirus outbreak in the Four Corners area was attributed to Native Americans, and even labeled the “Navajo disease” by media at the time (Person et al., 2004). More recently, during the outbreak of severe acute respiratory syndrome (SARS) in 2012–2013, Asian-American communities, regardless of their country of origin, also experienced discrimination, and were expected to quarantine as a group, regardless of whether or not they were exposed to contaminated individuals (Person et al., 2004). Stigmatization has also occurred in non-immigrant populations in previous pandemics, such as the residents of the complex that was most affected by SARS in Hong Kong (Lee et al., 2005).

Humans have evolved with an aversion to disease, and to perceived vectors of disease. This set of protective behaviors motivated by the aversion to potential vectors has been called the *behavioral immune system* by Schaller et al. (2003) and has since been referred to as the *parasite-stress model*. While these mechanisms may have served to reduce risk of infectious disease in ancestral social environments, they have historically come at the expense of intergroup relations (Schaller et al., 2003). Because infectious disease is not always apparent, and may indeed be asymptomatic in certain carriers, the behavioral immune system works by encouraging people to avoid unfamiliar outgroup members. Managing disease threat does not happen solely by avoiding or attending to the specific individuals who are affected by an illness, but by generalizing the perceived illness threat to the entire group, including those who are not afflicted, thereby attributing stereotypes of dirtiness and disease prevalence to these groups. The consequences include prejudiced attitudes rooted in feelings of fear and disgust toward these groups, inevitably engendering discrimination, such as exclusionary policies and violent attacks (Schaller and Neuberg, 2012). Indeed, priming participants to perceive greater disease prevalence increased prejudice toward both Black and white Americans (O'Shea et al., 2020), and experimentally manipulated perceptions of immunization among immigrants has the potential to reduce prejudice toward immigrants (Huang et al., 2011).

While the occurrence of a disease within an outgroup can lead to generalized prejudice toward that outgroup, disease does not always precede discrimination. Often, due to established structures of racism, outgroups are dehumanized in such a way that they are represented as carriers of pestilence and disease (e.g., Lawson, 2009). This framing has frequently been used against the same marginalized groups, including refugees, immigrants, racialized and ethnocultural minorities, and religious minorities (e.g., Steuter and Wills, 2009; Haslam and Loughnan, 2012; Esses et al., 2013; Utych, 2018), regardless of whether or not there is an actual disease in play. Furthermore, dehumanization is embedded in larger-scale, institutional, discrimination. Racism comprises many layers; at the systemic-level, racism is the structural disadvantage of racialized, religious and ethnocultural minorities (Krieger, 1999; Paradies, 2006; Feagin and Bennefield, 2014; Krieger et al., 2017). As a result of being socialized in these systemically racist structures, people develop racial biases, with stereotyped perceptions, prejudiced attitudes and feelings, and discriminatory behavior (Fiske, 1998). These manifestations can be obvious or subtle, as well intentional and unintentional (Sue et al., 2007). In the context of disease, a feedback loop can be observed where discriminatory policies, such as racial housing segregation, against minorities who have been dehumanized and stereotyped as dirty and prone to disease lead to greater vulnerability and spread of infectious diseases (Acevedo-Garcia, 2000). Often, the behavioral immune system, systemic racism, and dehumanization processes operate hand in hand (Hodson et al., 2014).

The COVID-19 pandemic has engendered, exposed, and exacerbated discrimination against minorities in several key ways: (1) prejudice and aggression toward minority groups associated with the disease (Gover et al., 2020); (2) disproportionate detrimental impacts of COVID in minority communities who are subject to preexisting discriminatory health inequities (Krieger et al., 2020); (3) increased vulnerability of minorities that are overrepresented in frontline and essential services (Saint-Girons et al., 2020), as well as communities in isolation, incarceration, displacement and occupied territories (Alemi et al., 2020; Santos et al., 2020); and (4) structural disadvantage in treatment access and vaccine distribution (Meyer, 2020; Power et al., 2020).

Anti-Asian racism rapidly increased after the onset of COVID-19 (e.g., Gover et al., 2020, Rich, 2020). The terms “Wuhan Virus” and “Chinese Virus,” have been used extensively (White, 2020) despite the World Health Organization's recommendation to not name diseases after regions or ethnic groups (World Health Organization, 2015), given that it is stigmatizing (e.g., Albader, 2020; Augustyn and Prazmo, 2020; De Costa, 2020; Su et al., 2020). COVID-19 has clearly been associated with East Asians both in their countries of origin and in the diaspora and has generalized to other groups that share similar phenotypic traits. East and Southeast Asian communities and individuals have been targeted globally with hate speech, including slurs, dehumanization, and slanderous, stereotyping comments about Chinese cuisine (Albader, 2020; Hu et al., 2020; Tahmasbi et al., 2021). Anti-Asian hate-crimes reports also spiked since the beginning of the pandemic in North America (Edara, 2020; Gover et al., 2020; Kotyk, 2020; Tessler et al., 2020; Tsai et al., 2020), Europe (Gao and Sai, 2020; Pellegrino, 2020; Velásquez et al., 2020, preprint) and Australia (Furlong and Finnie, 2020). In India, people from northeastern states with more phenotypically East Asian features have been discriminated against and attacked (Haokip, 2020). Even within East Asia, anti-Chinese prejudice was observed, specifically, in places like Hong Kong (Chung and Li, 2020) and South Korea (Albader, 2020).

In addition to East-Asians, Italians have been stigmatized in Europe after the country was severely affected by the virus (Pellegrino, 2020). Religious minorities have also been targeted as causes of the coronavirus. “Alt-right” groups in the US (Teter, 2020) and Canada (Currie, 2020) blamed Jews for the pandemic, and increased anti-Jewish discourse was observed in online spaces (Woodyatt, 2020). Increased anti-Muslim bias was reported in India with the onset of the pandemic, most notably with conspiratorial rumors blaming Indian Muslims for the spread of coronavirus (Mukherjee, 2020; Zajaczkowska, 2020). Within China, African migrant communities were blamed for the disease, and experienced an increase in anti-Black racism, such as evictions and refused services (Castillo and Amoah, 2020; Human Rights Watch, 2020).

Racism has also disproportionately impacted minority communities that were already vulnerable to health complications due to pre-existing inequities. The constant experience of systemic racism (i.e., segregation, food deserts, policing and criminalization, exclusion from employment and healthcare services) and of racism in everyday interactions (i.e., racist slurs, attacks, microaggressions, etc.) across generations inevitably harms and weakens the physical and mental health of minority group members (Krieger, 2014; Krieger et al., 2017). This health disadvantage creates disparities in the prevalence of serious chronic illnesses and greater mortality rates in racialized and ethnic minority groups (Pascoe and Smart Richman, 2009; Paradies et al., 2015). As a result of the prevalence of pre-existing health conditions in disadvantaged populations, members of such groups were more vulnerable to developing severe or fatal complications from COVID-19 (Krieger et al., 2020).

Black populations were among the most affected communities in terms of infection, hospitalization, severity of cases and mortality, notably in the US, Canada, and Britain (Egede and Walker, 2020; Gaynor and Wilson, 2020; Jain et al., 2020; Krieger et al., 2020; Public Health England, 2020). Other ethnic minority groups such as Latin-Americans (Krieger et al., 2020; Yearby and Mohapatra, 2020), South Asians and East Asians (Public Health England, 2020) have also been disproportionately impacted. In India, minority castes and tribes have been among the most vulnerable (Acharya and Porwal, 2020). Indigenous peoples in North America, Brazil, Australia and New Zealand have a higher risk of exposure, and have been diagnosed with more severe and fatal COVID-19 cases as a result of several factors, including health inequities that predate the pandemic, higher levels of frontline exposure, poverty, homelessness, displacement, overcrowding, and food and water insecurity (Arriagada et al., 2020; Furlong and Finnie, 2020; McLeod et al., 2020; Polidoro et al., 2020; Power et al., 2020; Saint-Girons et al., 2020; Yashadhana et al., 2020).

Often, these health inequities and treatment barriers exist because one's marginalized racialized or ethnic minority group is also subject to class marginalization, making access and affordability of resources and treatment challenging (e.g., Acharya and Porwal, 2020; Egede and Walker, 2020; Saint-Girons et al., 2020). Another way that class marginalization intersects with racism is through economic and employment disparities, where racialized and ethnic minorities face exclusion from higher status and higher income professions and are therefore segregated and relegated to lower-income and lower-status employment (Darity Jr, 2003; Sørensen, 2004; Armstrong et al., 2008). Minorities are therefore more likely to be exposed to COVID-19 because they are overrepresented in essential and frontline jobs and services, in prison systems, as well as in areas experiencing conflict and war, occupation and displacement. Racialized and ethnic minorities populate frontline and essential services, including healthcare and social services, agriculture, and food industries; workers in these sectors have been in more contact with patients and potentially infected peoples through their labor (Krieger, 2020). Migrant workers, indigenous peoples, and ethnic minorities around the world, including in India (Kesar et al., 2020; Sengupta and Jha, 2020), North America (Evans, 2020; Krieger, 2020; Saint-Girons et al., 2020), and Brazil (Teixeira, 2020) have been working in low-income and high-risk jobs, such as domestic labor, agriculture, transportation, and healthcare support. Another exposure risk for Indigenous peoples in North America has been the presence of others entering indigenous territory during the pandemic. This includes white, upper-middle class tourists (Leonard, 2020), as well as workers in resource extraction, since governments included mining industries in the category of essential services, prioritizing industry profit over Indigenous health (Bernauer and Slowey, 2020).

Ethnocultural minorities were already overrepresented in prisons due to structural disadvantage, including disproportionately elevated policing, criminal sentencing and incarceration of minority populations, and of Black, Indigenous and Latin-American communities in particular (Maynard, 2017; Mesic et al., 2018; Chartrand, 2019; Jahn et al., 2020). Incarcerated people are another marginalized segment of the population that experiences health inequities within a social institution that already poses higher risks of illness and infectious diseases, even those that are treatable (e.g., tuberculosis). Racialized and ethnocultural minorities in the prison system are among those with the highest rates of COVID-19 infection due to negligent safety and sanitation practices, overcrowding and scarce resources (Akiyama et al., 2020; di Giacomo et al., 2020; Gulati et al., 2020; Hagan et al., 2020; Lemasters et al., 2020; Santos et al., 2020). This has been the case in the US (Macmadu et al., 2020), the country with the world's largest incarcerated population (Akiyama et al., 2020), in Australia (Stewart et al., 2020), Brazil (Santos et al., 2020), and in countries with migrant detention prisons such as the US and Canada (Treisman, 2020).

Minority populations in displacement, conflict zones and occupied territories have also been at increased risk for contracting COVID-19. Refugees in camps are more vulnerable to the spread of COVID-19 due to lacking sanitation measures and basic infrastructure, greater population density, little to no secure housing, and greater health vulnerabilities due to injury and trauma (Alemi et al., 2020). This has been the case for Rohingyan refugees in Bangladesh (Islam and Yunus, 2020), Syrian refugees in different parts of West Asia and Europe (Kassem and Jaafar, 2020; Moawad and Andres, 2020), and refugees from South and Central America and the Caribbean (Brito, 2020). Refugees are also at risk of incarceration, which then places them at greater risk of exposure through the prison systems, or of deportation, which increases the risk of COVID-19 exposure and transmission across borders (Brito, 2020). The Nagorno-Karabakh War has led to additional losses due to COVID-19 (Kazaryan et al., 2020). Bombardments, destruction of houses, schools and hospitals, and the resulting displacement of peoples contributed to very high rates of COVID-19 in Armenia. Palestinians have also been more vulnerable to COVID-19 due to already limited resources, medical supplies, and infrastructure (Hammoudeh et al., 2020); in addition, Palestinians who normally worked in Israel have lost their livelihood due to the lockdown (Newman, 2020).

Discrimination is experienced by minorities through the lack of accessibility to treatment, as a result of segregation and exclusionary infrastructure. Marginalized minorities have limited access to healthcare, including medical care for COVID-19 (Evans, 2020; Polidoro et al., 2020; Yashadhana et al., 2020). In countries without universal healthcare, like the US, economically disenfranchised minority groups face economic barriers to receiving treatment (Spronk, 2020). Culturally-appropriate and adapted services are lacking, and can prevent minorities from seeking and benefiting from these services (Saint-Girons et al., 2020). Some Indigenous nations in the US are not even legally eligible to receive healthcare and are excluded from life-saving treatment as a result (Power et al., 2020). Other vulnerable populations such as refugees and undocumented peoples have difficult access to health services overall, which has also translated to difficulties receiving treatment for COVID-19 (Moawad and Andres, 2020). In addition, many healthcare services were already known to treat minority groups unfairly and cruelly (Goodman et al., 2017; Shingler, 2020). These bigger systemic disadvantages have contributed to minorities' historically justifiable distrust in a healthcare system that was designed to best serve dominant groups (Farquharson and Thornton, 2020; Iacobucci, 2020; Warren et al., 2020; Yearby and Mohapatra, 2020).

The disparities in access to healthcare and treatment are related to how the COVID-19 vaccines end up being distributed. The WHO has sponsored a COVID-19 Vaccine Global Access Facility (COVAX) to ensure equitable distribution of the vaccine globally (World Health Organization, 2020). However, many richer countries (e.g., the US, Canada and Britain) have already negotiated private deals with pharmaceutical companies for early access to a number of vaccines outside of the WHO (Meyer, 2020); whether these vaccines will be distributed equitably between countries, and within a given nation to minorities who have already been disadvantaged before and throughout the pandemic, is an ongoing concern.

## Individual Differences Shaped by Cultural Context

Cultural psychologists insist that neither mind nor behavior can be understood outside their sociocultural context. How people behave is based on: (1) their beliefs, even when they behave in ways contrary to professed beliefs; (2) the behaviors they observe in others, where these behaviors are interpreted in light of their beliefs (and thereby inferring the beliefs of others); and (3) the behaviors they believe others expect of them (Ryder et al., 2011, 2020). For example, a person may maintain disinfecting habits because they are conscientious, but nonetheless their practices are also influenced by public health announcements and what has normatively been defined as “good hygiene,” what they observe their neighbors doing (or ignoring), habitual religious practices that mandate cleanliness, sufficient resources to purchase extra hand sanitizer, and so on.

Individual differences such as levels of optimism were previously studied cross-culturally in the context of SARS. Ji et al. (2004) asked Chinese-Canadian and Euro-Canadian participants to estimate the risk of being infected during the SARS outbreak in Toronto, and the risk of an average person being infected. Participants from both groups overestimated their overall chances of getting infected while at the same time underestimating their risk of getting infected when comparing themselves to the average person. Chinese-Canadian participants reported more optimism, but were also more likely to take precautionary measures than were Euro-Canadians. Moreover, although the former group reported more inconveniences from the pandemic, they also reported more positive changes resulting from SARS (such as more appreciation for life and health, and feelings of being closer to family and friends), even though the experience of SARS itself was negative (Ji et al., 2004). This cyclical reasoning, differing from linear models of “cause and consequence” attributed to Western mindsets, had previously been observed in different studies comparing Chinese and European American populations (Ji et al., 2001). Even when studied cross-culturally, the widespread notion of individual differences, such as personality traits, is epistemologically rooted in the “Western” conceptualization of the self as an independent entity (Ryder et al., 2014). Here, we will briefly review studies on individual differences that were published in the context of COVID-19. However, we call for a reflection on how to integrate the cultural perspective into this debate, which we will explore in our last section.

### Personality Traits

Personality traits, in particular those of the “Big Five,” have been studied in the context of the pandemic, as an attempt to associate individual tendencies with behavioral responses to COVID-19. Across different countries, higher conscientiousness was linked to taking more precautionary measures and preparedness (e.g., stocking food), stronger feelings of insecurity in public spaces, and greater likelihood of keeping up with the news (Aschwanden et al., 2020; Asselmann et al., 2020; Carvalho et al., 2020). Openness-to-experience was linked to less intense feelings of insecurity in public, and extraversion was associated with less social distancing and sheltering in place, as well as shorter estimates for the duration of the pandemic (Aschwanden et al., 2020; Asselmann et al., 2020; Carvalho et al., 2020; Götz et al., 2020). Greater agreeableness was associated with stronger compliance with governmental recommendations, more precautionary measures, news attentiveness, and higher levels of trust in physicians (Asselmann et al., 2020; Götz et al., 2020; Qian and Yahara, 2020; Zajenkowski et al., 2020). Cross-cultural similarities suggest, to the extent that personality models can be mapped across different cultures, that these four traits are more homogeneously associated with similar behavioral outcomes in the countries considered by these studies. Nonetheless, in the case of neuroticism, cultural influences might have mediated the relation between this trait and behavioral responses in different countries. High neuroticism was correlated with fewer precautions in the United States (Aschwanden et al., 2020) and in Germany (Asselmann et al., 2020), but not in Japan (Qian and Yahara, 2020). However, to our knowledge, no study has yet explored potential explanations for the particular interplay between this trait and cultural context.

Personality traits associated with the dark triad (narcissism, Machiavellianism and psychopathy) have also been explored. Modersitzki et al. (2020) and Zajenkowski et al. (2020) found correlations between dark triad traits with the underestimation of the risks imposed by the pandemic. Additionally, *collective narcissism* may be particularly relevant to the emergence of nationalism under pandemic conditions (Bieber, 2020; Su and Shen, 2020; Woods et al., 2020). Collective narcissism involves a strong identification with one's own perceived group, accompanied by feelings of collective entitlement, unrealistic beliefs about the group, and outgroup hostility as a reaction to perceived threat (Zemojtel-Piotrowska et al., 2021). This trait has been classified into two types: agentic (unrealistic beliefs about the group's competence or dominance) and communal (unrealistic beliefs about the group's helpfulness or tolerance) (Nowak et al., 2020). Although collective narcissism is ultimately an individual trait, it is ingrained within and reinforced by broader political polarization and nationalist ideology. In Poland, Zemojtel-Piotrowska et al. (2021) observed that the agentic (but not the communal) form of collective narcissism was related to more perceived COVID-19 threats, and that such perceived threat mediated the relationship between collective narcissism and positive attitudes toward the European Union, as well as negative attitudes toward China. Both agentic and communal collective narcissism were negatively correlated with preventive behaviors such as washing hands, disinfecting objects, and staying home (Nowak et al., 2020). In the US and the UK, collective narcissism was associated with dissemination of conspiracy theories related to COVID-19 (Sternisko et al., 2020, preprint), which we will explore in more detail below.

### Attitudes and Beliefs

Individual attitudes and beliefs about the risks, prevention, and even the existence of COVID-19, are influenced by cultural factors, and motivate behaviors that affect the risks of contagion. Pre-symptomatic individuals contaminated with the novel coronavirus can spread it for up to four days before developing any symptoms (Christakis, 2020), and sudden spikes in cases are often attributed to “super-spreaders,” who are (knowingly or unknowingly) contaminated with the virus and participate in public gatherings, infecting disproportionally more people (Bruns et al., 2020). These gatherings may include parties, sport events, religious ceremonies, weddings, and funerals (Aherfi et al., 2020; Dave et al., 2020; Shanahan, 2020; Yasir, 2020). People who hold beliefs such as the non-existence of COVID-19, or who are skeptical about the dangers that the pandemic poses to society, are more prone to becoming a super-spreader.

Compliance with government recommendations has also been linked to individual beliefs about the effectiveness of the precautionary measures to protect oneself and the community, the dangers to one's own health (Clark et al., 2020), and the expectations about how official restrictions will develop (Bodas and Peleg, 2020; Briscese et al., 2020). Governments that were perceived as organized, consistent, and knowledgeable were more trusted during the pandemic (Han et al., 2021), but trust in the government was shown to be less relevant than beliefs about the efficacy of precautionary measures (Wong and Jensen, 2020), and more relevant to the tendency to underestimate the risks of the pandemic (Clark et al., 2020). In Italy, one of the first countries to adopt stay-at-home policies, residents reported being more likely to decrease self-isolation efforts if they were negatively surprised by an extension of the lockdown (Briscese et al., 2020). In Israel, 94% of individuals were willing to comply with quarantine recommendations if they were guaranteed financial support from the government for eventual lost wages, whereas only 57% expressed willingness to quarantine otherwise (Bodas and Peleg, 2020). In countries where the government denied the existence or severity of the pandemic, such as Brazil and the US (Anderson, 2020; Phillips, 2020; Reuters, 2020), and countries where the government actively promoted pseudoscience as a treatment, such as Tanzania and Madagascar (Resnick, 2020), trust in the government resulted in the decrease of efficient precautionary measures against COVID-19.

### Conspiracy Theories and Cultural Values

Although restricted to particular sociocultural contexts, research has linked individual differences to the likelihood of adhering to COVID-19 conspiracy theories (Biddlestone et al., 2020; Jovančević and Milićević, 2020; Uscinski et al., 2020). In some cases, distrust in authorities is historically linked to institutional abuse practiced against specific minority groups (e.g., the Tuskegee Study performed by the US government on Black patients; see Centers for Disease Control Prevention, 2021), which we will not discuss in the present review.

COVID-19 conspiracy theories typically consist of claims that the perils of the pandemic have been exaggerated by political groups, that the novel coronavirus was created in a laboratory and released as a bioweapon (Uscinski et al., 2020; van Bavel et al., 2020), or that COVID-19 vaccines will change one's DNA (Goodman and Carmichael, 2020). In Europe and North America, cellphone towers that would allegedly transmit coronavirus were damaged and destroyed (Chan et al., 2020; Valiante, 2020). In Brazil, conspiracy theories about China led 50% of Brazilians to reject the possibility of receiving the Chinese Sinovac vaccine before it was even available in the country (Amâncio, 2020; Londoño et al., 2020; Valle, 2020). In North America, COVID-19 fueled the surfacing of the far-right conspiracy theory movement QAnon, which has been causing ruptures between people and their families (Blackwell, 2020).

In a study on cultural orientation and COVID-19, Biddlestone et al. (2020) analyzed the correlations between engagement in COVID-19 conspiracy theories, intention to reduce the spread of the virus, and cultural orientation toward collectivism and individualism. The authors differentiated horizontal individualism (the view of the self as autonomous, with preference for equality) and vertical individualism (view of the self as autonomous, with acceptance of inequality) and horizontal and vertical collectivism (view of the self as part of the collective, with emphasis on equality and acceptance of inequality, respectively), following the approach of Triandis and Gelfand (1998). Vertical and horizontal types of collectivism—but neither type of individualism—were positively associated with intentions to engage in social distancing. Vertical individualism, in particular, was correlated with adherence to COVID-19 conspiracy theories (Biddlestone et al., 2020). Other research has similarly found that individualistic worldviews are associated with less perceived risks from COVID-19, besides lowering intentions to adopt preventive measures against the transmission of the virus (Dryhurst et al., 2020; Huang et al., 2020).

In the US context, psychological predispositions to reject expert information and accounts of major events, partisan and ideological motivations, and religiosity are also predictors of beliefs in COVID-19 conspiracy theories (Uscinski et al., 2020). Belief in conspiracy theories also correlated with increased levels of fear and pessimism, while optimism and higher levels of general trust correlated with more engagement in preventive behaviors (Jovančević and Milićević, 2020).

### Mental Health and Well-Being

In a study performed in 26 countries, Kowal et al. (2020) concluded that people in countries and areas that were more severely affected by COVID-19 were more stressed. Additionally, higher levels of stress were reported by women, people staying with children, single people, younger people, and people with lower levels of education. However, higher levels of education seemed to correlate with higher reports of depressive symptoms and lower levels of life satisfaction at the beginning of the pandemic, particularly in the US (Wanberg et al., 2020). In different countries, health professionals, particularly nurses, have experienced increased levels of stress (Barzilay et al., 2020; Ilczak et al., 2020).

Individual differences in optimism and intolerance of uncertainty under COVID-19 have been shown to impact mental well-being as well as adaptation to the new circumstances imposed by the pandemic. Higher levels of optimism are correlated with better work routine adjustment both in professionals who are working from home (Biron et al., 2020) and in health care professionals (Zhang et al., 2020). The cultural context also plays a role in individual adjustment, notably collectivism and collective optimism (a shared optimism about a group), as these characteristics favor the sense of mutual obligation in times of crisis and effective coping strategies, like positive reappraisal (Biron et al., 2020).

Finally, intolerance of uncertainty (difficulty accepting feelings and thoughts related to uncertain scenarios) can also impact mental health. Cultural contexts differ widely in terms of how much uncertainty one is accustomed to, the amount of uncertainty believed to be tolerable, and available practices that help mitigate the adverse effects of uncertainty. The pandemic led to a worldwide increase in uncertainty, due both to fear of a hitherto unknown virus combined with the sudden changes in routines, social interactions, financial and professional security, and grieving rituals. Here, we should consider the extent to which new uncertainties and changes damage people's access to basic needs, as well as what “uncertainty” means to different populations. For instance, the political pressure to re-open commerce in different parts of the world has more impact on individuals who are not privileged enough to “move on with life as usual” (Manderson and Levine, 2020); thus, the duration of the uncertainty experienced by certain groups can be strikingly different.

Individual experiences of distress are heavily influenced by the degree to which a cultural and socioeconomic context enables, rewards, or prevents adaptation to change. In the US, workers in industries like technology were able to switch into a home-office work routine, keep high-paying jobs and move to more affordable and less crowded regions, having reported decreased levels of stress (Peyser, 2020). In contrast, people with low socioeconomic status are less likely to be able to self-isolate due to life circumstances (Templeton et al., 2020), and those with lower-income employment who were already in a riskier socioeconomic bracket were more likely to lose their jobs entirely or be forced to keep working in high-exposure conditions in order to save their livelihoods (Rollston and Galea, 2020).

## Theoretical and Practical Implications

In this review, we have argued that the impact of infectious diseases on different groups and societies is highly responsive to collective beliefs and behaviors, which interact with biological characteristics of these diseases in a way that favors or inhibits transmission. Such beliefs and behaviors, combined with local norms and values, constitute complex narratives captured in cultural scripts, and are influenced by the intensity and reach of exchanged information across individuals, families, and communities, and by broader political and socioeconomic dynamics. We examined how these influences played out at different levels (cross-society, within-society and individual) in 2020, in the context of the first major pandemic to emerge in a deeply globalized world whose social and political dynamics are profoundly intertwined with the use of technology and the fast transmission of information.

We have adopted concepts from cultural and cultural-clinical psychology in our analysis. To some extent, our approach suggests non-immediate applications to clinical science, as the sociopolitical circumstances discussed are dynamically linked to mental health. More importantly, we believe it also provides important theoretical insights and tools to understand how the mutual constitution of mind and culture affects the way we conceive, react to, and think about health and illness more broadly in psychology and adjacent disciplines. From our perspective, the course of every disease, even when the etiologies are unambiguously biological, is contingent on how individuals, groups and societies understand it, experience it and respond to it given local cultural scripts.

We also urged an integration of political and socioeconomic dynamics into the cultural psychology debate, in the context of infectious diseases and more broadly. Mental health conditions are determined by subjective experiences and sociocultural norms that interact with such experiences (e.g., stigmatization, cultural expectations, access to basic needs and social support networks); infectious diseases should be similarly understood. Dynamics of power, privilege and oppression constitute, as discussed, looping effects that mutually reinforce individually- or collectively-held beliefs, as well as subjective experiences of the pandemic and epidemiological outcomes. Marginalized ethnocultural groups are now at greater risk for COVID-19 exposure and infection; the barriers to treatment then spur the spread further into these disadvantaged communities. Even individual differences, when manifested at the collective level and enabled by local cultural and political factors, feed into loops that reinforce or prevent experiences of discrimination and racism.

Examining looping effects can yield insights about which stages of the loops can be interrupted to attenuate the impact of infectious diseases. Lack of trust in the healthcare system, for instance, can be reinforced by several factors, such as the exclusion of people who are unable to follow recommendations due to limited access to resources (Templeton et al., 2020), absence of culturally-sensitive communication in healthcare settings, systemic discrimination of specific populations, and even the unsuitability of medical models that were conceived in Western contexts and are incompatible with patients' individual understanding and experiences of health and illness. Each of these scenarios would require different kinds of institutional interventions, and should be analyzed in their particular sociopolitical and cultural contexts. Additionally, we believe that mental health professionals can benefit from and be sensitized by this discussion, not only because these effects might manifest in the context of diagnoses and treatment, but also because individual experiences of health and illness, under COVID-19 or otherwise, are permeated by such sociopolitical and cultural influences.

Culture interacts with biological factors in the context of infectious diseases: the novel coronavirus has a similar transmissibility as the virus that causes SARS. However, SARS's higher fatality rate slowed down its transmission, since a larger proportion of infected individuals manifested symptoms quickly and died before transmitting the virus. Conversely, the incubation period of the novel coronavirus is longer, and so pre-symptomatic individuals expose and infect others over a longer period before they realize that they are infectious (Christakis, 2020). Thus, the same set of behaviors can produce different outcomes depending on the biological characteristics of the virus, which in turn feeds into or contradicts the pre-existing shared beliefs about the virus. These beliefs do not exist in a vacuum; cultural and political contexts enable different sets of beliefs, and at the collective level those are directly related to epidemiological outcomes. In our perspective, effective public health messaging would be strategized around local shared beliefs about the pandemic, which can be assessed by methods already established in cultural psychology and related fields.

A cultural psychology perspective on COVID-19 encourages a deeper conversation about the profound extent to which psychological science is rooted in Western conceptualizations of the self, and has not completely overcome its historical tendency to individualize behavior and the experience of suffering, by attributing it more to dispositional tendencies than to situational contexts. The scientific community constitute, in itself, a group with its own cultural biases and social structures (Hoshmand, 1996), that are permeated by larger power dynamics. The pandemic amplified phenomena that are at the root of several psychopathological conditions (e.g., social isolation, prolonged financial insecurity, stigmatization), all of which have yet to be formally and thoroughly taken into account in psychopathology research, and incorporated into clinical training. Future research in health, clinical and medical social sciences can benefit from the sensitization to the interplay between culture and intersectionalities that are not always obvious or reducible to theoretical models: individual experiences should not be simplified into mere interactions between dispositional tendencies and stereotyped cultural characteristics.

The pandemic also expanded our understanding of the enormous impact of racism to the mental health of racialized and marginalized peoples. Anti-Asian aggression and hate speech (Cheah et al., 2020; Litam, 2020; Tessler et al., 2020), racism targeting Muslims and other religious minorities (Mukherjee, 2020), and other forms of discrimination have impacted minority communities around the world, as can be observed through increased depression, anxiety, trauma, and other mental health issues, and at rates higher than those of majority groups (Ghandour et al., 2020; Mukherjee, 2020). Moreover, due to their overrepresentation in frontline and essential services, racialized and ethnic minorities are more likely to experience multiple role strain and stress as they juggle parenting and work without social or economic support (Walters, 2020).

Minority communities have demonstrated resilience and community support in the face of these obstacles. Indigenous communities in Canada, for instance, developed their own infrastructure to attend to their own community members' health and well-being in response to the existing systemic barriers to food, water and healthcare (Power et al., 2020). In Brazil, where *favelas* (urban informal settlements), were severely affected by the pandemic (Airhihenbuwa et al., 2020), independent and internally-organized movements have been facilitating access to food and protective goods such as hand sanitizers, besides providing orientation about COVID-19 and autonomously monitoring symptomatic individuals (Goldenbaum and Galante, 2020). Overall, we cannot simply examine this disease as a one-size-fits-all stressor, but as a disease that interacts with existing structures and inequities to create disproportionate suffering and responses in those who are already disadvantaged.

Additionally, we believe that our perspective equips us with a better understanding of how we can increase the cultural sensitivity of public policies and science communication in the context of pandemics. Incorporating cultural scripts poses challenges such as the identification of locally-relevant cultural factors and the need to develop cross-cultural measurement instruments (Hruschka and Hadley, 2008; Kohrt et al., 2009). Culture influences individuals' beliefs and worldviews about societal dangers, which is reflected in their commitment to different forms of social organization (Kahan, 2012). Therefore, awareness interventions should take into consideration these variables, as well as local language needs, cultural values, relational mobility, tightness-looseness, health inequities and discrimination, trust in the government and the perception of science by different groups. This is congruent with Airhihenbuwa et al. (2020), which proposes a cultural model of public health messaging composed of three domains: cultural identity, relationships and expectations, and cultural empowerment. They argue that such messaging should be inclusive of multiple “cultural logics.”

We noted that at the societal level, higher levels of collectivism and tightness seem to predict better public engagement in protective behaviors and, as consequence, more efficacy in battling the virus (Cao et al., 2020; Jiang et al., 2020). Some authors propose that, in looser and more individualistic countries, public health policies should be stricter and coordinated across all societal levels to reduce the impact of COVID-19 (Cao et al., 2020), which would require the incorporation of cultural awareness into intervention strategies.

Science communication initiatives should also attend to local cultural scripts and political dynamics, as both factors influence responses to fear, uncertainty and change. Although the level of COVID-related fear might increase the voluntary compliance with official recommendations, it seems to also decrease the effect of collectivism (and consequently, compliance) on such engagement (Huang et al., 2020). Intense experiences of fear also have long-term effects over mental health and, during the pandemic, have triggered suicides in some countries (Satici et al., 2020), which suggests that the promotion of collectivism and the action for the common good is a better strategy than tailoring propaganda to evoke fear. Moreover, the engagement of trusted local leaders can increase the effectiveness of collectivistic public health messages (van Bavel et al., 2020), and the promotion of empathy for vulnerable groups (e.g., older adults, racialized and ethnic minorities, marginalized groups) seems to increase compliance with social distancing regulations more broadly in the population (Jiang et al., 2020). From a risk-perception perspective, Wong and Jensen (2020) argue that, in extreme situations such as the pandemic, a “defensive pessimism” is a good strategy to prepare people for long-term changes. Thus, increasing the awareness of the risk that the pandemic imposes, as well as avoiding the communication of unrealistic expectations about official restrictions, could better prevent disruptions in voluntary engagement in public health recommendations, which is compatible with the findings we discussed (e.g., Briscese et al., 2020).

In countries where COVID-19 has been highly politicized, the development of effective interventions is more challenging. Partisan-motivated reasoning leads individuals to support or oppose policies according to the endorsement of their favorite political parties (Bolsen et al., 2014). Ideally, policies to limit the spread of the coronavirus would benefit from open support from opposing political parties. However, in contexts where political leaders and parties have systematically denied the risks posed by COVID-19, societies might benefit more from the initiative of other organizations, such as NGOs, science communication collectives and media outlets. Particularly in the US, where TV channels have been openly partisan, political polarization was intensified during the pandemic (Green et al., 2020) and politicians featured in newspaper coverage more often than experts (Hart et al., 2020). For these audiences, the engagement of third-party, apartisan organizations and local leaders in the transmission of credible, evidence-based information and public health recommendations might be more advantageous.

Anti-vaccination movements constitute another challenge and operate in the context of cultural scripts, and were already considered one of the top threats to global health before the pandemic (World Health Organization, 2019). The emergence of COVID-19 fueled the preexisting trend of massive propagation of anti-vaccination fake news, as COVID-19 has brought not only drastic changes to people's routines and wellbeing, but also led to an unprecedented use of social media (Holmes, 2020; Nielsen, 2020). Social media's algorithms are optimized to increase engagement, which creates a territory that is conducive to the propagation of misinformation (Caulfield, 2020) and anti-science digital populism (Monteiro, 2020). Unsurprisingly, evidence shows that individuals search for medical advice on the internet and consider pseudoscientific claims as equally valid as evidence-based opinions (Kata, 2012). “Alternative facts” propagators manipulate information to increase the perception that the fabricated facts are plausible and of public relevance and utilize tactics such as the promotion of incredulity toward experts and polarization against a common “enemy,” which can be the government or the scientific community depending on local shared beliefs. The fear and anxiety triggered by COVID-19, combined with the local perception of science, and the preexisting trend toward trust in governments across the globe (Alves and Mutsvairo, 2019; Svolik, 2019) create a scenario that is conducive to misinformation and conspiracy theories, which requires thoughtful and thorough cultural awareness among science communicators and policy-makers. Future research could examine the role of cognitive biases on the interplay between vaccine attitudes and individual differences related to conspiratorial thinking under pandemic situations in different cultural contexts.

We are dealing with a unique combination of new complex social dynamics at the global level amidst a sanitary crisis. Understanding these dynamics from a cultural psychology perspective that is integrated with a broader discussion on the complex factors that shape the social lives of infectious diseases can be informative on how to better communicate and promote protective measures, help people cope with their current realities, and promote inequity repair and solidarity with marginalized communities.

## Author Contributions

AR and MY conceptualized the manuscript. RB reviewed the literature and wrote the first draft. MY and AR contributed specific sections to the first draft, all authors contributed substantially to the final version and approved the submitted version.

## Funding

We acknowledge the financial contribution of Concordia University's COVID-19 Established Researcher Support, awarded to AR.

## Conflict of Interest

The authors declare that the research was conducted in the absence of any commercial or financial relationships that could be construed as a potential conflict of interest.

## Publisher's Note

All claims expressed in this article are solely those of the authors and do not necessarily represent those of their affiliated organizations, or those of the publisher, the editors and the reviewers. Any product that may be evaluated in this article, or claim that may be made by its manufacturer, is not guaranteed or endorsed by the publisher.
